# Clinical Grade Production of Wilms’ Tumor-1 Loaded Cord Blood-Derived Dendritic Cells to Prevent Relapse in Pediatric AML After Cord Blood Transplantation

**DOI:** 10.3389/fimmu.2020.559152

**Published:** 2020-09-25

**Authors:** Maud Plantinga, Vania Lo Presti, Colin G. de Haar, Ester Dünnebach, Alejandro Madrigal, Caroline A. Lindemans, Jaap Jan Boelens, Stefan Nierkens

**Affiliations:** ^1^Center for Translational Immunology, University Medical Center Utrecht, Utrecht, Netherlands; ^2^Pharmacy Department, Cell Therapy Facility, University Medical Center Utrecht, Utrecht, Netherlands; ^3^UCL Cancer Institute, Royal Free Hospital, London, United Kingdom; ^4^Princess Máxima Center for Pediatric Oncology, Blood and Marrow Transplantation Program, Utrecht, Netherlands; ^5^Stem Cell Transplant and Cellular Therapies, Department of Pediatrics, Memorial Sloan Kettering Cancer Center, New York, NY, United States

**Keywords:** dendritic cells, vaccine, cord blood, immunotherapy, transplantation, good manufacturing practice

## Abstract

Hematopoietic cell transplantation (HCT) is a last resort, potentially curative treatment option for pediatric patients with refractory acute myeloid leukemia (AML). Cord blood transplantation (CBT) results in less relapses and less graft-versus-host disease when compared to other sources. Nevertheless, still more than half of the children die from relapses. We therefore designed a strategy to prevent relapses by inducing anti-AML immunity after CBT, using a CB-derived dendritic cell (CBDC) vaccine generated from CD34+ CB cells from the same graft. We here describe the optimization and validation of good manufacturing practice (GMP)-grade production of the CBDC vaccine. We show the feasibility of expanding low amounts of CD34+ cells in a closed bag system to sufficient DCs per patient for at least three rounds of vaccinations. The CBDCs showed upregulated costimulatory molecules after maturation and showed enhanced CCR7-dependent migration toward CCL19 in a trans-well migrations assay. CBDCs expressed Wilms’ tumor 1 (WT1) protein after electroporation with *WT1*-mRNA, but were not as potent as CBDCs loaded with synthetic long peptides (peptivator). The WT1-peptivator loaded CBDCs were able to stimulate T-cells both in a mixed lymphocyte reaction as well as in an antigen-specific (autologous) setting. The autologous stimulated T-cells lysed not only the WT1+ cell line, but most importantly, also primary pediatric AML cells. Altogether, we provide a GMP-protocol of a highly mature CBDC vaccine, loaded with WT1 peptivator and able to stimulate autologous T-cells in an antigen-specific manner. Finally, these T-cells lysed primary pediatric AML demonstrating the competence of the CBDC vaccine strategy.

## Introduction

Acute myeloid leukemia (AML) is the second most prevalent leukemia (15%–20%) with a 5-year survival rate in children of ~50%–60% (1). As a last treatment resort, patients with certain high-risk cytogenetic features (e.g. FLT3-ITD without concomitant NPM1) (2) and therapy-refractory patients or patients with relapsed AML who achieve CR2 after re-induction therapy are eligible for allogeneic hematopoietic cell transplantation (allo-HCT) (3).

Nevertheless, despite the better survival rates, a clear unmet need to further reduce the relapse rates and enhance survival post HCT remains for high risk AML patients. Maintenance, preemptive or salvage therapeutic strategies have been explored to prevent relapse post allo-HCT, e.g. FMS-like tyrosine kinase 3 (FLT3) inhibitors, Isocitrate Dehydrogenase (IDH) Inhibitors, Hypomethylating Agents and HDAC Inhibitors and more recently also cellular therapies (4). A promising cellular therapeutic strategy to induce specific anti-AML immunity early after HCT is dendritic cell (DC) vaccination. In the past, mainly monocyte-derived (mo)DC based vaccines or peptides were tested in clinical trials (5). However, the numbers of monocytes and antigen-presenting cells, in particular DC, are very low after transplant which very much limits the potential success of vaccination strategies at these early time points. The use of Cord blood (CB) as stem cell source for allo-HCT specifically in children, has important advantages over either bone marrow (BM) or mobilized peripheral blood (PB) stem cells, since CB produce a more powerful graft-versus-leukemia effect (6–9); the T-cell reconstitution (in particular CD4+ cells) is excellent in absence or with personalized dosing of anti-thymocyte globulin (ATG) and contains mostly naïve T-cells that seem to transform rapidly into effector T-cells (10, 11). The reduced risk of developing graft versus host disease (GvHD) (10, 12, 13) and viral reactivation after HCT without serotherapy have supported the use of CB as a cell source for transplantation (10, 12, 14, 15). In addition, this provides an autologous source for stem cell-derived DC for vaccination after HCT. We previously designed a protocol which enables to generate DC from the 20% fraction of a CB unit (the 80% fractions are used to engrafting the patient). In short, enriched CD34+ CB-derived stem cells were expanded for 1 week and differentiated for an additional week, followed by maturation using an inflammatory cytokine mix and thereafter electroporated with *Wilms’ tumor 1 (WT1)* mRNA (16). The protocol generated CBDC in sufficient numbers as used in previous moDC vaccination studies (total dose in adults 0.1–20×10^6) (17–19).

We used WT1 as a tumor-specific target as it is overexpressed in the majority (>80-90%) of patients with AML, including cell-cycle quiescent AML stem cells located in the BM (20). In addition, younger subjects with AML showed more frequent recurrent mutations in WT1 than adults (21). In a recent study, Chapuis et al. inserted a WT1-specific TCR (C4) into Epstein-Bar virus-specific donor CD8+ T-cells from healthy donors and infused these cells prophylactically post-HCT into 12 adult AML patients with encouraging results (22). This study supports the rationale to stimulate WT1-specific T-cell responses post-HCT to reduce relapse rates.

We here describe the translation of a preclinical DC culture protocol to a good manufacturing practices (GMP)-setting using a closed bag culture system. We compared the use of peptivator, consisting of lyophilized long peptides covering the complete sequence of the human WT1 protein, with the introduction of a GMP-grade *WT1*-mRNA construct for electroporation to ensure the expression of the full length WT1 protein. The potency to select and stimulate WT1-specific T-cell from CB and subsequently lyse primary pediatric AML cells by these T-cells were studied as functional outcomes.

## Methods

### CB Collection and CD34 Isolation

Umbilical cord bloods (CB) were obtained from the Anthony Nolan Cord Blood bank (UK) with inform consent in accordance with the Human Tissue Act 2004, the Human Tissue Regulations 2007 and the HTA’s Code of Practice in the United Kingdom. The CB was shipped using a dry-shipper and stored in the vapor phase of a dedicated controlled and continuously monitored liquid nitrogen tank to the production site until thawing. CB was thawed using thaw-medium (CliniMACS^®^ buffer with HSA, MgCL_2_ and Pulmozyme) in a transfer bag. Cells were collected in tubes and after washing twice, the cells were labeled with magnetic-anti CD34 beads, washed to remove unbound beads and thereafter the labeled cells were resuspended in a transfer bag and enriched using the CliniMACS device (Miltenyi Biotec).

### CBDC Culture

The two-step protocol consists of an expansion and differentiation phase and is performed in the GMP-accredited cell therapy facility of the University medical center Utrecht. For the first week of expansion 5×10^4^ CD34^+^ cells/ml are cultured and the second week 1×10^5^ CD34^+^ cells/ml are cultured in X-VIVO 15 (Lonza) supplemented with Flt3L (50 IU/ml), SCF (50 IU/ml), IL-3 (80 IU/ml) and IL-6 (2,400 IU/ml). In the 3^rd^ and 4^th^ test run 5-10% human serum is added to increase cell yield. After washing, the cells are differentiated at 1×10^5^ cells/ml in X-VIVO 15 containing 5% human serum and supplemented with Flt3L (50 IU/ml), SCF (20 IU/ml), GM-CSF (280 IU/ml) and IL-4 (240 IU/ml) for another week (23). To induce maturation the cytokines used for differentiation plus an inflammatory cytokine mix, a combination of IL-1beta (700 IU/ml), IL-6 (2,400 IU/ml), TNFα (350 IU/ml), and PGE2 (1 µg/ml) from Pfizer, was added to the culture for 24 h with or without WT1 peptivator (0,12 nmol/ml). The WT1 peptivator contains a pool of lyophilized overlapping oligopeptides, covering the complete sequence of the WT1 (Swiss-prot Acc.no.P19544). Overall, cells are thawed, enriched, expanded and differentiated in bags. Between different culture phases, e.g. expansion, differentiation and maturation, cells were collected in 50 or 250 ml centrifuge tubes, spun down and resuspended in a small volume new X-VIVO 15 in a sterile flask, including the cytokines of either expansion, differentiation or maturation. Cell counting was performed using trypan blue. Medium was added to obtain the correct concentration and the cell suspension was transferred to a MACS^®^ Cell Differentiation Bag (CDB; Miltenyi) *via* a luer lock system. All GMP-grade recombinant human cytokines, growth factors and WT1 Peptivator^®^ were obtained from Miltenyi Biotec. In total 10 CB donors were used, five for the optimization runs and five for the validation runs. At the end of the procedure WT1-loaded CBDCs were frozen per 10–15×10^6 cells/vial in freezing medium (20% X-VIVO 15, 20% human serum and 10% DMSO) at −196°C. For further use the vial was thawed in 50% human serum and 50% X-VIVO 15 medium at 37°C.

### Electroporation and WT1 Detection

Mature CBDC were loaded with *WT1*-encoding mRNA (AmpTec GmbH) by electroporation (EP) as previously described (24), with minor modifications. Briefly, 5–10×10e6 cells in 200 µl OptiMEM media were transferred to a 4-mm electroporation cuvette (Bio-Rad, Hercules, CA, USA) and electroporated with 9,5 ug RNA by a time constant pulse of 300 V for 7 ms using the Gene Pulser Xcell device (Bio-Rad). After EP the cells recovered for 4h in the medium used to mature the cells. Next, WT1 expression was determined. Cells were washed with FACS buffer (PBS containing 2% BSA (Sigma-Aldrich) and 0.1% sodium azide (NaN3, Sigma-Aldrich) prior to a 15 min incubation at room temperature (RT) in the dark with a fixable viability dye (Thermo fisher). Next, cells were washed and fixed/permeabilized (eBioscience) for 30 min at 4°C. After washing with permeabilization buffer, anti-WT1 (F6-H2; (Dako) or purified mouse IgG1κ (MG1-45; Biolegend) as isotype control and FcR Blocking (Miltenyi Biotec) was added for 15 min at RT in de dark. The cells were washed again with permeabilization buffer before adding the F(ab’)2 Anti-mouse IgG PE antibody (eBioscience) for 15 min at RT in the dark. Cells are resuspended in FACS buffer and analyzed using a FACS CantoII (BD) flow cytometer. Analysis was performed using FlowJo software (Tree Star, Inc.).

### Quality Control: Microbiology

The cells were carefully monitored by morphology during the entire culture period, and sterility testing of vaccines was performed. Mycoplasma was analyzed by the Nucleic acid amplification technique (NAT) by Polymerase Chain Reactions (PCR). Endotoxin was assessed by the kinetic chromogenic limulus amoebocyte lysate (LAL) test (Lonza).

### DC Phenotype

To assess DC phenotype, cells were collected from the bags and washed twice FACS buffer. Thereafter, the cells were incubated at 4°C and stained with the appropriate antibody combinations. Antibodies used for flow cytometry for DC phenotype and purity include: HLA-DR, CD45, CCR7 (from Biolegend), CD11b, CD15, CD16, CD33, CD56, 7-AAD, CD14, CD3, CD83, CD 80, CD11c (all from BD), CD14, CD19, and CD117 (from Beckman & Coulter). For intracellular (IC) stainings, cells were washed with FACS buffer after surface staining and treated with Cytofix/Cytoperm (BD), according to the manufacturer’s protocol, followed by 30 min of incubation at 4°C with the following Abs for the non-DC fraction: cytoplasmic Myeloperoxidase (MPO; Dako). Multiparameter analysis was performed on a FACS Canto II or LSR Fortessa II (BD) flow cytometer. Dead cells were excluded by scatter gating. Analysis was performed using DIVA (BD) or FlowJo software (Tree Star, Inc.).

### Transwell Migration Assay

*In vitro* migration assays were performed using 24 transwell (3 µm pore size) plates (Greiner). In brief, 400.000 CBDCs in 200 ul culture medium (X-VIVO 15 with 5% human serum) were plated in the upper compartment. Culture medium, either alone or supplemented with 250 ng/ml CCL19 (R&D systems), was added to the lower compartment. After 2 h, DCs were collected from the lower compartment. The cells are washed and stained for CD11c, HLA-DR and CD83 and analyzed using flow cytometry in a fixed volume. Counts measured by flow were used to validate migration.

### Mixed Leukocyte Reaction

CD3 cells were purified from allogenic CD34^-^ cells using anti-CD3 magnetic microbeads (Miltenyi). These responder CD3 cells (1x10^6^/ml) were then labeled with cell trace violet (5 µM; Invitrogen), and cocultured with matured CBDCs (2x10^5^/ml) as stimulator cells. Unstimulated cell trace violet-labeled cells served as negative control. After 4 or 5 days, cells were stained with CD3, CD8, CCR7 (Biolegend), CD4 (Ebioscience), CD45RO (BD), and CD69 (Sony) and analyzed using a FACS LSR Fortessa (BD). T-cell proliferation analysis was performed using the proliferation tool in flowjo (Tree Star,Inc.), providing the division index, the average number of cell divisions of one cell in the original population.

### WT1 Antigen Presentation

*WT1 mRNA* electroporated CBDCs in combination with WT1 peptivator, or peptivator loaded DCs alone or DC alone were cocultured with 1×10e6/ml of our previously developed HLA-A2–restricted WT1-specific T-cell clone recognizing the WT1 37-45 (VLDFAPPGA) epitope at a DC-to-T-cell ratio of 1:1 for 5 h in the presence of Golgi-stop (1/1500; BD). T2-cells loaded with/without WT1-peptivator (Miltenyi) were used as a respectively positive and negative control. The T-cells were subsequently stained for CD3 (Biolegend), CD8 and extracellular expression of LAMP-1 (BD) and intracellular IFNg (Ebioscience) expression, followed by flow cytometry–based analysis.

### Isolation, Expansion, and Identification of WT1-Specific T-Cells

CBDC culture was performed as described above with 1 week of expansion. To isolate antigen-specific T-cells from cord blood the CD34- population was thawed and resuspended in RPMI-1640 medium (Gibco) supplemented with 5% human serum (Sanquin). A maximum of 1x10e8 CD34- cells were stimulated by the addition of 1 ug/ml CD28 monoclonal antibody (Miltenyi Biotec) and WT1 peptivator (Miltenyi Biotec). Antigen-reactive CD8+ T-cells were isolated after 24 h using a CD137 microbead kit (Miltenyi Biotec) according to manufacturer’s instructions (25). Antigen-reactive T-cells were cocultured with autologous matured CBDC in X-Vivo15 medium (Lonza) supplemented with 5% human serum (Sanquin) and 15 ng/ml IL-21 (Miltenyi Biotec) at a maximum ratio of 1:100 effector:APC. Plate 1 ml/well in a 24-wells plate. After 24–48 h half of the medium was changed every other day with fresh medium supplemented with 5 ng/ml IL-7, 5 ng/ml IL-15, and 10 ng/ml IL-21 (all Miltenyi Biotec). After 10 days of enrichment and expansion, T-cells were stained to identify tetramer+ T-cells. Tetramer panels were generated according to established protocol (26), consisting of 8 WT1 peptides; WT1(27), WT1(37), WT1(126), WT1(187), WT1(225), WT1(235), WT1(242), WT1(436), CMV-tetramer is used as control. Cells are pre-treated with 50 nM dasatinib (VWR international), to stabilize the TCR on the surface, for 30 min at 37°C prior to staining with the tetramer panel. Cells were washed once with FACS buffer and tetramer panel was prepared by adding approximately 0,1 ug per peptide:MHC complex to brilliant violet staining buffer (BD). Incubated 15 min at 37°C. Without washing a 5x stock of the following antibodies: anti-CD8 (BD), anti-CD4, anti-CD14, anti-CD16, anti-CD19 (all life technologies) and fixable viability dye (Ebioscience) is added to the sample and incubated for 30 min on ice. Cells were washed twice with FACS buffer, resuspended in FACS buffer and multiparameter analysis was performed on a FACS LSR Fortessa (BD) flow cytometer. Analysis was performed with FACS Diva software (BD).

### Killing Assay

Primary AML cells from the bone marrow were obtained from SKION biobank from the Princess Máxima Center for Pediatric Oncology, obtained after broad informed consent and selected for HLA-A2+ donors. These target AML cells were thawed, washed and suspended in RPMI + 10% human serum. AML cells were confirmed to express HLA-A2 and WT1 levels and after two washes with serum free PBS labeled with 0.4 µM cell trace violet (CTV; Invitrogen), diluted to 1x10e5/ml and cocultured with WT1-specific T-cells as effector cells in a 10:1 and 40:1 ratio when indicated. HLA-A2+ WT1-expressing cell-line 697 (derived from a patient with acute lymphoblastic leukemia) was used as a positive control and K562 ((A2-WT1+) derived from a patient with chronic myeloid leukemia) and unstimulated CTV-labeled cells serves as a negative control. After 18–24 h, cells were incubated with 7AAD (final dilution 1:200; BD) for 15 min at RT. 10.000 beads (Beckman Coulter) were added just prior to measurement for quantification to measure a fixed amount per sample. All samples were measured on CantoII (BD). Percentage of lysis was calculated by 100% (viable target cell count/viable target cell count of target only condition × 100%).

## Results

### GMP Production Optimization

**Figure 1:**
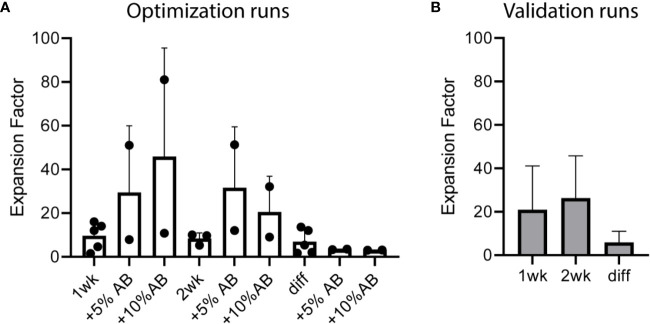
Cell expansion over time in cord blood (CB)-culture of the five optimization and five validation runs performed under good manufacturing practice (GMP) conditions. Expansion factor (EF) expansion week 1 means total cells week 1 divided by CD34+ number at start; EF week 2 means total cells week 2 divided by week 1; EF diff is total cells after differentiation divided by expansion week 2. **(A)** EF of the five optimization runs including five or 10% human serum during expansion and an additional week of expansion (runs 3–5). **(B)** EF of the five validation runs. Two weeks of expansion (week 1 and week 2) followed by 1 week of differentiation (diff), which contains 5% human serum. Error bar represents SD.

A total of five preclinical GMP production runs were performed for the translation of the preclinical culture protocol (16) to generate CBDC into a GMP-production process and to further optimize the procedure. CD34+ cells were enriched from the CB using CliniMACS providing 0.73×10^6 (0.18–2.6×10^6) CD34+ cells with a viability of ≥70% and a purity of 72%–92%, and subsequently expanded in culture bags. Run 1 and 2 showed lower expansion using CD34-enriched cells from a frozen CB unit compared to fresh CB. Adding AB serum during the first week of expansion and the addition of a second week of expansion were tested in the next runs to achieve sufficient numbers of cells (**Table 1**). Next, the expanded cells were differentiated toward DCs and reference vials were obtained. The expansion factor (EF), calculated by the number of live cells (×10^6) divided by the number of cells at the start before the medium change, was high in the expansion phase, increased slightly adding serum, and lower in the differentiation phase in the optimization runs (**Figure 1A**). After 1 week expansion followed by differentiation, roughly 12 and 65×10^6 DCs (run 1 and 2) were obtained, compared to 3, 388, and 443×10^6 DCs (run 3–5) with 2 weeks expansion. Two weeks expansion is necessary to ensure sufficient myeloid precursors to differentiate toward DCs (at least 50×10^6 cells) from each batch of cord blood. This expansion capacity is confirmed in the validation runs (**Figure 1B**). The addition of serum increased the yield to some extent during expansion, but decreased the total number of mature DCs in some batches (**Table 1**). It was therefore omitted from the final protocol. From these preclinical test runs we concluded that the final protocol would be a 3-week protocol without addition of human AB serum to the first week.

**Table 1:** Preclinical good manufacturing practice (GMP) optimization runs.

	CD34+ (x10^6)	EF expansion week 1	EF expansion week 2	EF differentiation week 1	total EF	total vaccine cells (x10^6)	% CD83 CBDC	Summary protocol
**Run 1**	0.38	16	x	2	32	12	19	1 week exp
**Run 2**	2.6	4.6	x	5.4	25	65	52	1 week exp
**Run 3**	0.27	12	10	12	1,440	388	50	2 weeks exp
**Run 3 + 10%AB**	0.27	81	9	3	2,187	590	15	2 weeks exp + serum
**Run 4**	0.18	1.5	5.3	1.7	13.5	3	NA	2 weeks exp
**Run 4 + 5%AB**	0.18	7.9	51.3	3.5	1418	255	NA	2 weeks exp + serum
**Run 4 + 10%AB**	0.18	10.8	32.1	3.1	1,075	194	NA	2 weeks exp + serum
**Run 5**	0.24	14	9.7	13.6	1,847	443	39	2 weeks exp
**Run 5 + 5%AB**	0.24	51	12	3.3	2,020	485	33	2 weeks exp + serum

Five test runs from five donors were performed to test for sufficient number of mature CBDCs. Run 1 and 2 were subjected to 1 week expansion, for run 3–5, two weeks of expansion. In run 3–5 either 5% or 10% human serum (AB) was added to the expansion. CD34+ cells after enrichment using CliniMACS. EF dividing the total cell number by the cell number at the beginning of the week. % CD83 CBDC are the mature DCs of total cells at the end of the culture measured using flow cytometry. x= no 2^nd^ week of expansion in the first runs; EF, expansion factor; NA, not assessed; exp, expansion.

### GMP Production Validation

After completion of the procedure-optimization runs, we performed 5 additional GMP runs to validate the final protocol. 0.75×10^6 (0.34–1.24×10^6) CD34+ cells were subjected to expansion and differentiation, generating 235×10^6 (145–300×10^6) cells in the final product, i.e. sufficient to obtain the minimum of 50×10^6 cells for three rounds of vaccination and the appropriate amount of reference and retention vials. Data from these runs were used to check the feasibility to reach the release criteria for the final product.

**Figure 2:**
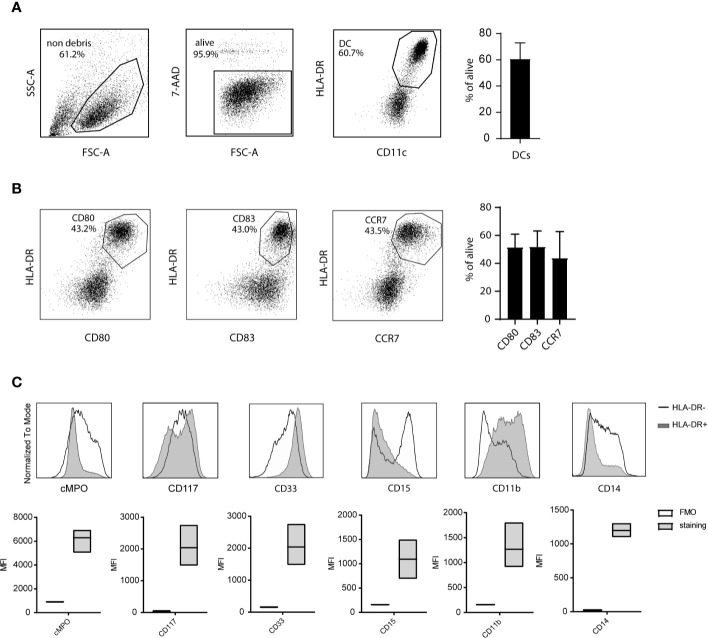
Phenotype of cord blood (CB)-culture. **(A)** Gating strategy for CB-derived dendritic cells (CBDCs) using flow cytometry. **(B)** Expression of costimulatory levels of CD80, CD83, and CCR7 expression on the 7-AAD- cells in the CB culture. **(C)** Myeloid expression profile within the HLA-DR negative (black line) and HLA-DR positive (CBDC; grey) population (histogram) or HLA-DR- compared to fluorescence minus one (FMO) staining (MFI boxplots) in the CB-culture after maturation. **(A, B)** represent 1 out of 5, the mean of five validation runs is calculated for **(C)**.

Next, the phenotype of the CBDC within the live gate was assessed by flow cytometry. The protocol of expansion, differentiation and maturation resulted in 60% (+/-10%) CD11c+ HLA-DR+ cells (**Figure 2A**). In the validation run CBDC were positive for the maturation marker CD80 (average 58% [43-70%]) and CD83 (average 56% [43-70%]) (**Figure 2B**). CC-chemokine receptor 7 (CCR7) was highly upregulated on CBDC (average 44% [20-67%]), enabling *in vivo* migration (**Figure 2B**). T- (CD3+), B- (CD19+), and NK-cells (CD16+CD56+) were measured in the CB culture after maturation in the 5 validation test runs: all T-, B-and NK-populations were below 0.1% (gating strategy in **Supplementary Figure 1**). The remaining cells in the culture are primarily myeloid precursors based on cytoplasmic expression of Myeloperoxidase (cMPO) and surface expression of CD33 and CD117. In addition, levels of CD14, CD15 and CD11b are detected in part of the HLA-DR negative fraction (**Figure 2C**).

Altogether, the GMP production protocol resulted in sufficient CBDCs with a highly mature phenotype and purity (release criteria ≥30% CD83/80+ DCs). These CBDCs are subjected to a variety of assays described to determine their potency.

### Migration and Allogeneic T-Cell Activation by CBDCs

**Figure 3:**
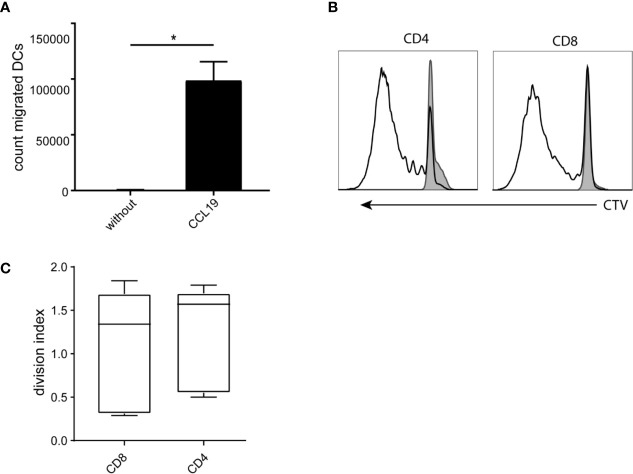
Functionality of the cord blood -derived dendritic cells (CBDCs). **(A)** Number of cells migrating toward a CCL19 gradient (or without as control) in a trans well system. **(B)** Histogram of dilution of Cell tracer violet of CD4 and CD8 CB T-cells stimulated with CBDCs (black) compared to T-cells alone (filled grey). **(C)** Average of either CD4 or CD8 T-cells that went into division after stimulation with CBDCs from a different donor. The division index is calculated using Flowjo after measuring cell proliferation by cell tracer violet dilution using flow cytometry. Statistics analyzed in GraphPad Prism using Mann-Whitney test. *= < 0.05. The experiments were performed with five different donors.

CBDCs express high levels of CCR7 on the surface, illustrating the capacity to migrate toward a CCL19 gradient. Indeed, significant migration of 60.000 cells on average was observed to the lower compartment in a transwell system, compared to an average of 560 cells in the absence of CCL19 (**Figure 3A**).

Next, as an outcome measure of DC functionality, allogeneic T-cell proliferation (mixed leukocyte reaction; MLR) was measured using T-cells from a different CB donor. CBDC induced CD4 as well as CD8 proliferation, with a division index of 1.2 and 1 respectively (**Figure 3B**), showing the capacity to stimulate T-cells in an antigen-independent manner (**Figure 3C**).

### Antigen Loading and Presentation

**Figure 4:**
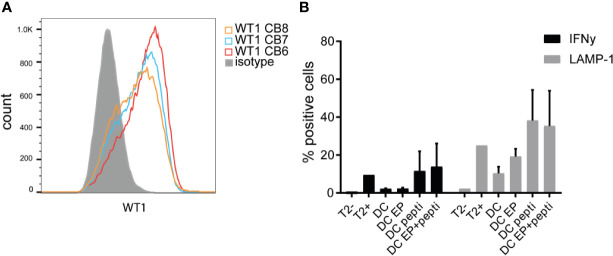
WT1 expression and presentation. **(A)** Intracellular WT1 levels measured by flow cytometry after electroporation of *WT1* mRNA compared to isotype control (grey). Validation runs 6–8 are shown. **(B)** Percentage of intracellular IFNg levels and extracellular LAMP-1 expression by WT1-specific CD8 T-cells activated by cord blood-derived dendritic cells (CBDC) electroporated with *WT1* mRNA (DC EP), loaded with peptivator (DC pepti), both (DC EP+pepti) or nothing (DC). T2 cells in the presence or absence of peptide served as controls. 2 CBDC donors were used. Statistical analysis is performed in GraphPad Prism using Kruskal-Wallis test, no significant differences.

We compared peptivator loading, 15-mers with a 12-mers overlap covering most of the WT1 protein, to electroporation (EP) of mRNA encoding for 2 major isoforms of the full-length WT1 protein. An *in vitro* T-cell avidity assay with different antigen concentration showed that the dose administered to the CBDC (1ug/ml/peptide) is sufficient for loading CBDC (**Supplementary Figure 2**). After EP, intracellular WT1 levels were detected on CBDCs using flow cytometry (**Figure 4A**). However, EP resulted in extensive loss of cells (**Supplementary Figure 3**). Next, these DCs were tested for their capacity to activate antigen-specific T-cells using a previously generated CB-derived WT1_37-45_ T-cell clone. Surface LAMP-1 expression and intracellular IFNg expression in the WT1-specific T-cell clone was measured after a coculture with either *WT1* mRNA electroporated DCs (WT1 EP DCs), WT1-peptivator-pulsed DCs or a combination of both. WT1 EP DCs showed minimal T-cell activation: 19% LAMP-1 and 2,3% IFNg expression, while loading with peptivator increased LAMP-1 and IFNg expression to, 21% and 38,2% respectively. The combination of loading strategies did not show an additional effect compared to peptivator-pulsed DCs alone (13,9 and 35,4%) (**Figure 4B**). Because of the low WT1 specific T-cell activation, in particular IFNg and high loss of cells in the procedure, EP was ommited from the final GMP production protocol.

### Autologous WT1 T-Cell Assay and AML Lysis

**Figure 5:**
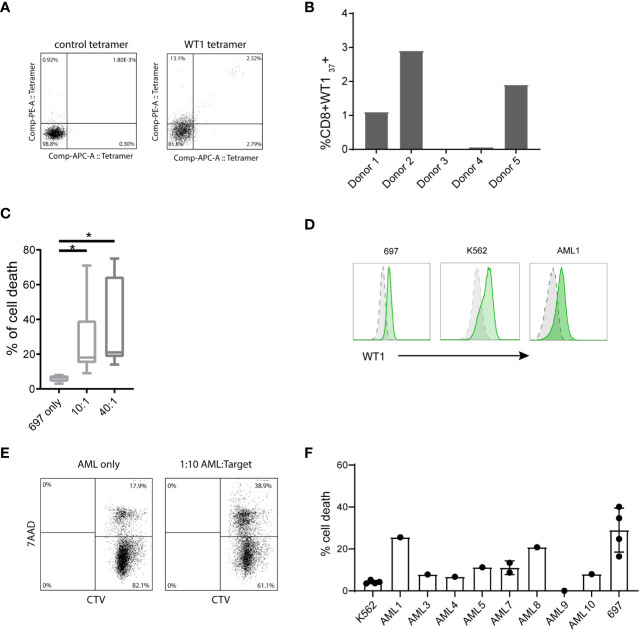
T-cell activation by cord blood-derived dendritic cell (CBDC) and subsequently acute myeloid leukemia (AML) lysis by CB WT1+ T-cells. **(A)** Gating example of WT1-pulsed T-cells, analyzed by either CMV tetramer (negative control) or WT1 tetramer. **(B)** Frequency of CD8+WT1_37_+ generated from naïve bulk T-cells stimulated by peptivator loaded CBDCs. **(C)** Percentage of cell death of HLA-A2+ WT1+ 697 cell line in the absence of T-cells or in a 10:1 or 40:1 ratio with CD8+WT1+ T-cells generated from different donors. Target T-cells were labeled with cell tracer dye (CTV) and lysis was assessed detecting % 7-AAD+ CTV+ target T-cells. **(D)** Example of intracellular WT1 levels (green) of 697, K562 and primary AML1 compared to isotype control (grey). **(E)** Gating of lysed target cells, pre-gated on CTV+. **(F)** Percentage of cell death of primary pediatric AML compared to K562 (negative control) or 697 as a positive control. Error bar represents multiple killing assays (2–4) and represents SD. Statistical analysis is performed in GraphPad Prism using Kruskal-Wallis test.

Next to allogeneic (WT1) stimulation, we tested the stimulatory capacity in an autologous assay. After overnight stimulation of the whole CB unit with WT1 peptivator, WT1-specific T-cells were enriched by CD137+ cell isolation and subsequently re-stimulated with autologous WT1 peptivator-pulsed CBDCs. These T-cells were expanded and characterized by tetramer staining. A few clones were detected of which one dominant HLA-A2; WT_37-45_ (VLDFAPPGA) of the 8 HLA-A2 tetramers measured (**Figure 5A**). In **Figure 5B** the relative contribution of the different T-cells in different donors is shown (**Figure 5B**). In most cases, the tetramer+ T-cells were FACS-sorted and further expanded. Some WT1+ T-cells were employed for a killing assay using a WT1-expressing cell line (697). Robust target cell lysis was observed in 20%–40% of the cells (**Figure 5C**). In addition, we added the T-cells to pediatric primary AML cells to observe primary AML lysis. WT1 is not measured routinely and therefore WT1and HLA-A2 (**Figure 5D** and **Supplementary Figure 4**) were stained in parallel. A variety of WT1 levels is observed (mean MFI 316 [25-713]). Lysis was observed in 5 out of 10 samples when compared to background levels of K562 (negative control) (**Figures 5E, F**). These CB-T-cells stimulated by CBDC suggested antigen specific AML cell lysis.

### Viability and Survival

**Figure 6:**
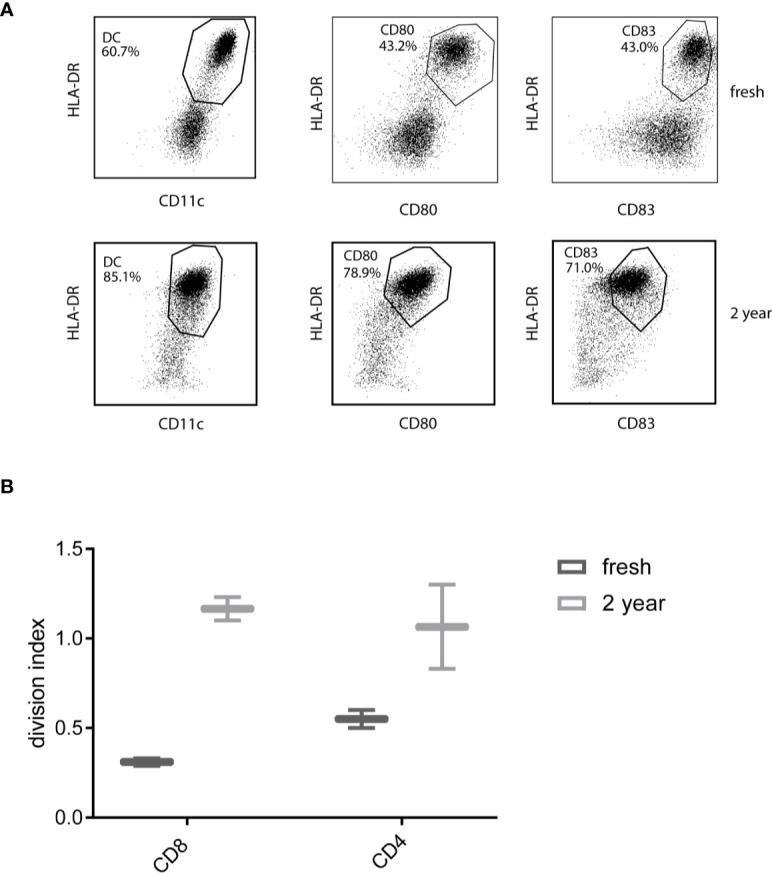
Phenotype and functionality after thawing. **(A)** Phenotype of 7AAD-cord blood-derived dendritic cells (CBDCs) after thawing a reference vial from the test runs either directly after generation or after 2 years. **(B)** Division index from a mixed leukocyte reaction (MLR) stimulated by directly thawed or CBDC thawed after 2 years. The T-cells used for the MLR are from a different donor, CBDCs from the same validation run.

Since CBDC will be frozen prior to the use for vaccination, at several time points the effect of cryopreservation is monitored. Less than two months, three months (not shown) and 2 years after manufacturing CBDC, cells were thawed and subjected to a MLR potency assay. The viability after 2 years was 73%. The CBDC showed expression of CD11c, HLA-DR, CD80 and CD83 (**Figure 6A**). The division index (the average number of cell divisions) in a MLR was comparable or even increased compared to the CBDC thawed ≤ 2months after freezing (**Figure 6B**). In conclusion, we are able to generate sufficient mature CBDC, that are not affected by cryopreservation, to use for vaccination after CBT.

### Release Requirements for CBDC Vaccine

**Figure 7:**
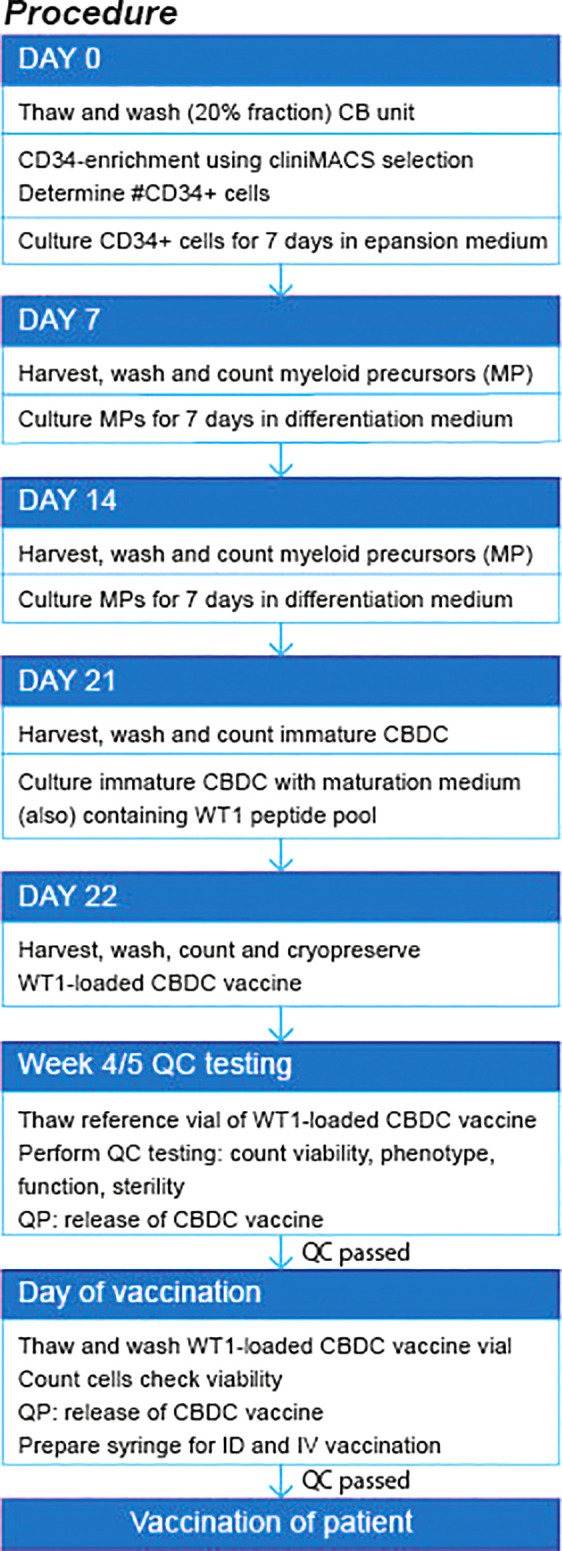
Flow diagram of production procedure. QP, quality personal; QC, quality control. ID, intradermal; IV, intravenous.

The GMP manufacturing process of the CBDC vaccine was optimized in five optimization runs followed by five validation runs. The first five optimization runs were used for translation and optimization of the preclinical culture protocol into a GMP-production process. The validation runs were all performed using the same protocol and provided sufficient information on the efficacy, variation and reproducibility of the manufacturing process, from frozen CB unit to thawed and ready to administer WT1-loaded CBDC vaccine (**Table 2**). Ultimately, sufficient number of viable cells required for vaccination (at least 5x10^6 cell/vaccination) will need to be present after thawing and washing of the cryopreserved product. Mature DCs are able to prime naïve T-cells and are potent T-cell activators. As such, the expression of the hallmark surface proteins of mature DC (≥30%), i.e. CD80 and CD83, should be present in the CB culture, while containing a minimum of contaminating cells, like T-, B- and NK-cells (≤5%). Furthermore, microbiological sterility of the thawed vaccine is secured using the reference vial, frozen in addition to the vaccine. All 5 validation runs met these release criteria as indicated in **Table 2** and finalized the CBDC protocol (**Figure 7**).

**Table 2:** Quality control testing results of final product n=5 donors (directly thawed reference vials of the validation runs 6–10).

Test of	Specifications	Runs				
		6	7	8	9	10
**Cell count**						
Total viable cells	≥5×10^6 for max dose	6.7×10^6	7.2×10^6	6.0×10^6	5.8×10^6	6.5×10^6
Viability	≥ 60% of total cells	76%	81%	79%	75%	78%
**Phenotype**						
Mature CBDC (CD83+ HLA-DR+)	≥ 30%	70%	50%	56%	50%	64%
Costimulation (CD80+ HLA-DR+)	≥ 30%	70%	47%	56%	49%	60%
**Lymphocyte contamination**						
B cells	≤ 5%	0.06%	0.12%	0.07%	0.09%	0.26%
T-cells	≤ 5%	0.02%	0.03%	0.01%	0.08%	0.19%
NK cells	≤ 5%	0.04%	0.12%	0.05%	0.35%	0.18%
**Microbiological control of cellular products**	Sterile (Ph.Eur.)	Yes	Yes	Yes	Yes	Yes

## Discussion

The DC vaccination field is advancing with implementation of state-of-the-art techniques enabling the selection of different sources of DCs, antigen loading strategies, and genetic modification of the cells (27). As relapse rates post-HCT are still relatively high in pediatric AML, we hypothesized that selecting and stimulating AML-specific T-cells by DC vaccination post-HCT could protect children from AML relapse. The patients will be vaccinated while in complete remission (CR) and the generation of anti-AML immunity will not be hindered by the suppressive tumor environment as observed in patients with a high tumor burden (28). Notably, DC vaccination in a minimal residual disease setting or in combination with surgery or chemotherapy, improved its efficacy in other studies (29, 30). DC vaccinations have been used in a large number of clinical studies, including those performed after allo-HCT, suggesting that DC vaccination post allo-HCT is safe (31–35). In all these studies the DCs were derived from monocytes. The use of CD34-derived DCs for vaccination has been limited to an autologous DC vaccination setting for the treatment of metastatic melanoma (36, 37). In addition, no DC vaccinations have ever been performed after CBT. Since the same graft is used for CBT and generation of DC, optimal DC-T cell communication is warranted. Moreover, CBDCs are loaded with peptides covering the majority of the WT1 protein, therefore no HLA restrictions and concomitantly no HLA-based selection of patients is necessary. The proposed combination is particularly suitable for children and young adolescents, since adult patients with high risk AML often receive transplants from HLA-matched sibling donors (38).

We previously showed in a preclinical study that CB stem cells can be expanded and differentiated into DCs resembling primary cDC2 cells (16, 39). In the work presented here, we translated the preclinical protocol into a GMP-grade culture protocol for the generation of an advanced therapy medicinal product. A major difference was the replacement of culture flasks with a closed bag system for cell expansion and differentiation. The DC phenotype and function was similar in the different plastics used for flasks/plates and bags (this manuscript and (16)), but fewer differences were observed between CB donors when using bags. The reason for this has not been studied but could relate to a different organization of the multilayers of cells (microenvironment) in the bags (40, 41). The cells showed expression of activation markers, migration and MLR capacity, and were able to stimulate WT1-specific cell lines and autologous CB-derived WT1 specific T-cells that subsequently acquired cytolytic capacity to kill primary AML blast. We showed that sufficient cells can be generated from a low number of isolated CD34+ CB stem cells for at least 3 vaccinations and that the product can be frozen and thawed without losing efficacy. Quality criteria for this product will include the number of viable cells (at least 5x10^6 cells/vaccination) present after thawing and washing of the cryopreserved product, the expression of CD80 and CD83 to show the DC have matured (≥30%), a minimum of contaminating cells, like T-, B- and NK-cells (≤5%), and microbiological sterility of the thawed vaccine using a reference vial. Since the product is made from a separately frozen compartment of the cord blood graft (20% fraction) and the product will be frozen again before administration to the patient, the generation can be outsourced to a manufacturing facility and shipped to the hospital where the patient received his/her HCT.

So far, we focused on WT1 as a target of pediatric AML. The selection of a tumor associated antigen remains a major challenge for the development of a CBDC vaccine. Neoantigens are of high interest, because of their high tumor specificity (42), but in contrast to, for example pediatric ALL, *de novo* AML is characterized by a very low burden of genomic alterations: more than one third of pediatric AML cases lacks any identifiable copy number alteration, and more than a quarter of the leukemia’s with recurrent translocations lacks any identifiable sequence or numerical abnormalities (43). WT1 is overexpressed in the majority (>80-90%) of patients with AML, including cell-cycle quiescent AML stem cells located in the BM (44) and relapses after CBT, which supports the choice of WT1 as a tumor target (45, 46). Although it has been suggested that consistent high-avidity WT1 T-cell responses could not be demonstrated in vaccination studies (47), others successfully isolated a high-affinity WT1-specific TCR (TCR_C4_) and reported the first positive results from the clinical trial using WT1 TCR-engineered adoptive T-cell (22). Moreover, vaccination with mRNA WT1-loaded moDC as a post-remission treatment in high-risk adult AML prevented or delayed relapse in 43% of the 30 patients included in the study (48–52). The increased survival correlated with the occurrence of WT1-specific T-cell responses, but unfortunately, not all patients responded. Recent results from a phase 2 trial of a multivalent WT1 peptide vaccine showed beneficial clinical outcomes that correlated with an immunological response in a non-transplant setting (53, 54).

We tested different antigen loading techniques: mRNA encoding the full length WT1 protein versus a mix of synthetic overlapping WT1 peptides. The exogenous delivered long peptide antigens will immediately be loaded on MHCII for presentation to CD4 T-cells, but will require uptake and delivery into the MHC class I cross-presentation pathway to stimulate CD8 T-cells

Peptide loading resulted in a better T-cell stimulation efficacy and in a significant reduction in the loss of CBDCs due to the transduction procedure. For these reasons we decided to include the peptide loading as it would simplify the GMP-procedure, increase the number of cells for vaccination while maintaining good efficacy.

In conclusion, with the purpose of developing a clinical grade DC vaccine from CB CD34+, we here established a GMP-protocol to generate sufficient CB-derived DCs in a culture bag for at least three vaccinations post-HCT. The CBDCs are highly mature, able to migrate and activate WT1-specific T-cells, which in turn lyse WT1+ cell lines and primary pediatric AML samples. These CBDCs may augment the reconstituting immune system toward anti-WT1 T-cell activity and thereby prevent relapse in refractory AML patients.

## Data Availability Statement

The original contributions presented in the study are included in the article/**Supplementary Material**; further inquiries can be directed to the corresponding author.

## Ethics Statement

The studies involving human participants were reviewed and approved by Nolan Cord blood bank. Full informed consent is obtained from expectant mothers in accordance with the Human Tissue Act 2004, the Human Tissue Regulations 2007 and the HTA’s Code of Practice in the United Kingdom. The patients/participants provided their written informed consent to participate in this study.

## Author Contributions

Conceptualization, MP, CH, and SN. MP, VLP, and ED performed the experiments. MP made the figures and wrote the manuscript. AM, CL, and SN critically reviewed the paper. AM provided CB. Supervision by JJB and SN.

## Funding

This research was funded by ZonMW/TAS 116003003 and KIKA nr 142.

## Conflict of Interest

The authors declare that the research was conducted in the absence of any commercial or financial relationships that could be construed as a potential conflict of interest.

## Supplementary Material

Supplementary Figure 1 Gating strategy of purity after the whole procedure. Reference vial from the 5 validation runs were used to perform purity staining using flow cytometry. 1 representative shown.

Supplementary Figure 2 T-cell avidity assay. Normalized IFNg production of WT1-specific T-cells stimulated by four different CBDCs, pulsed with different WT1-antigen concentrations (M).

Supplementary Figure 3 Loss of cell number after EP. Cell count before and after EP. 200x10^6 cells were electroporated for each sample.

Supplementary Figure 4 WT1 and HLA-A2 expression by primary AML samples. All obtained AML samples were measured using flow cytometry. (**A**) Mean fluorescent intensity (MFI) of intracellular WT1 levels of the different primary pediatric AML samples. WT1 positive AML samples were selected for the killing assay (**figure 5**) (**B**) MFI of HLA-A2 levels expressed in bars (left) or histogram (right) to show variable expression although selected from the database on genetically positive levels of A2.
