# Supplementary figures and images for: Clinical Grade Production of Wilms’ Tumor-1 Loaded Cord Blood-Derived Dendritic Cells to Prevent Relapse in Pediatric AML After Cord Blood Transplantation
Source: Front Immunol. 2020 Sep 25;11:559152. doi: 10.3389/fimmu.2020.559152 (PMC7546401; doi:10.3389/fimmu.2020.559152)

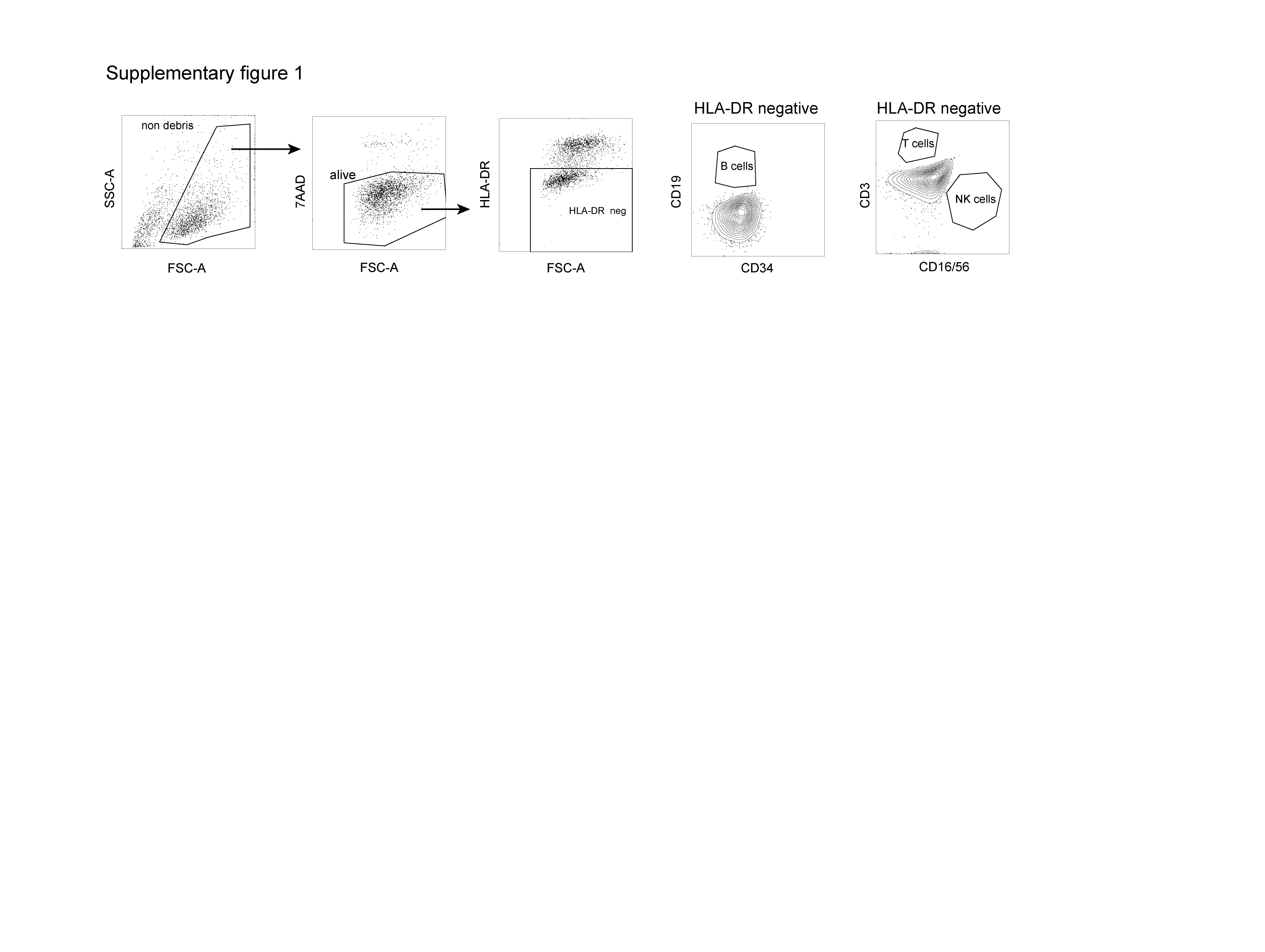

Supplement: Supplementary Figure 1 — Gating strategy of purity after the whole procedure. Reference vial from the 5 validation runs were used to perform purity staining using flow cytometry. 1 representative shown. [file Image_1.tif]

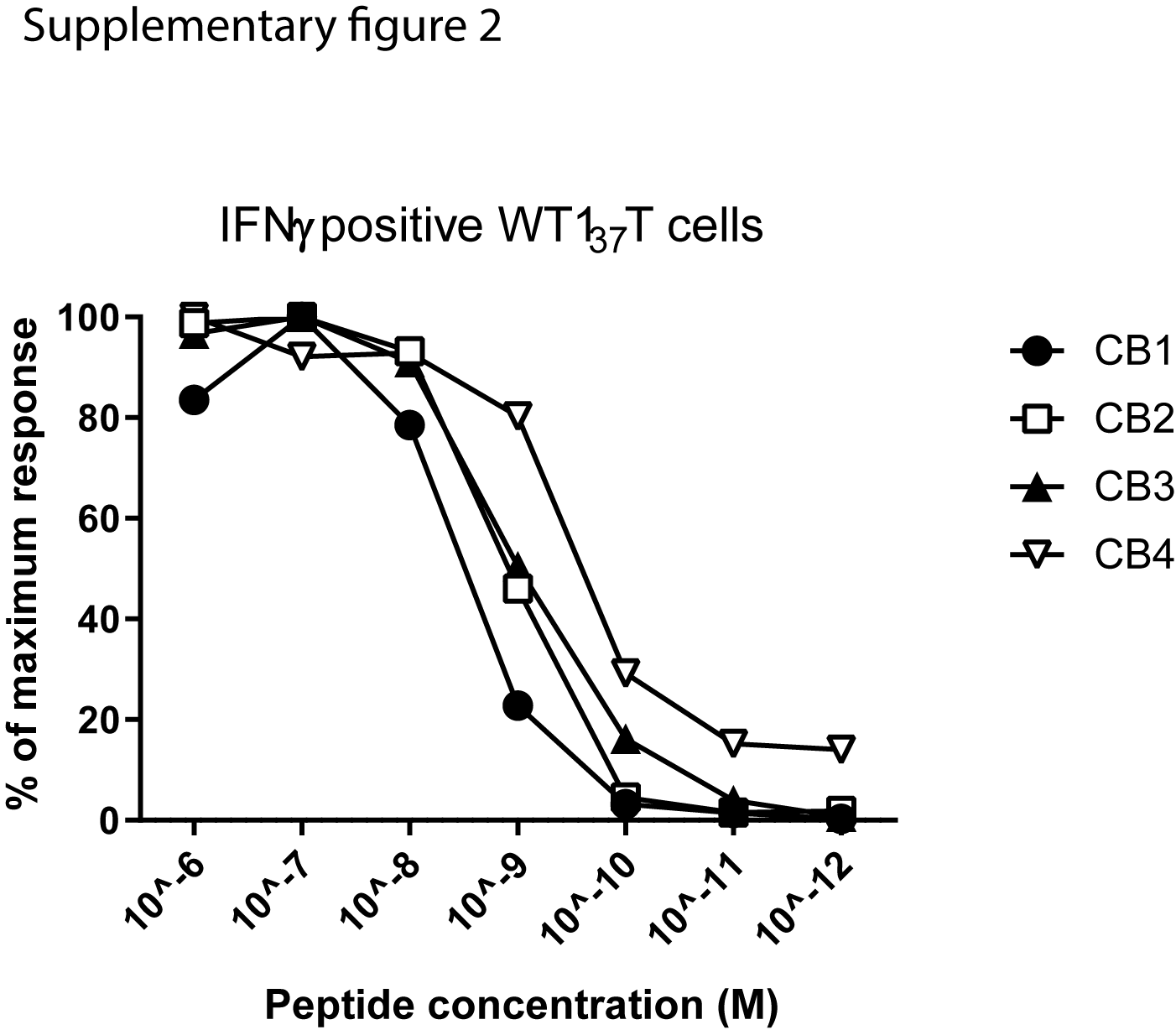

Supplement: Supplementary Figure 2 — T-cell avidity assay. Normalized IFNg production of WT1-specific T-cells stimulated by four different CBDCs, pulsed with different WT1-antigen concentrations (M). [file Image_2.tif]

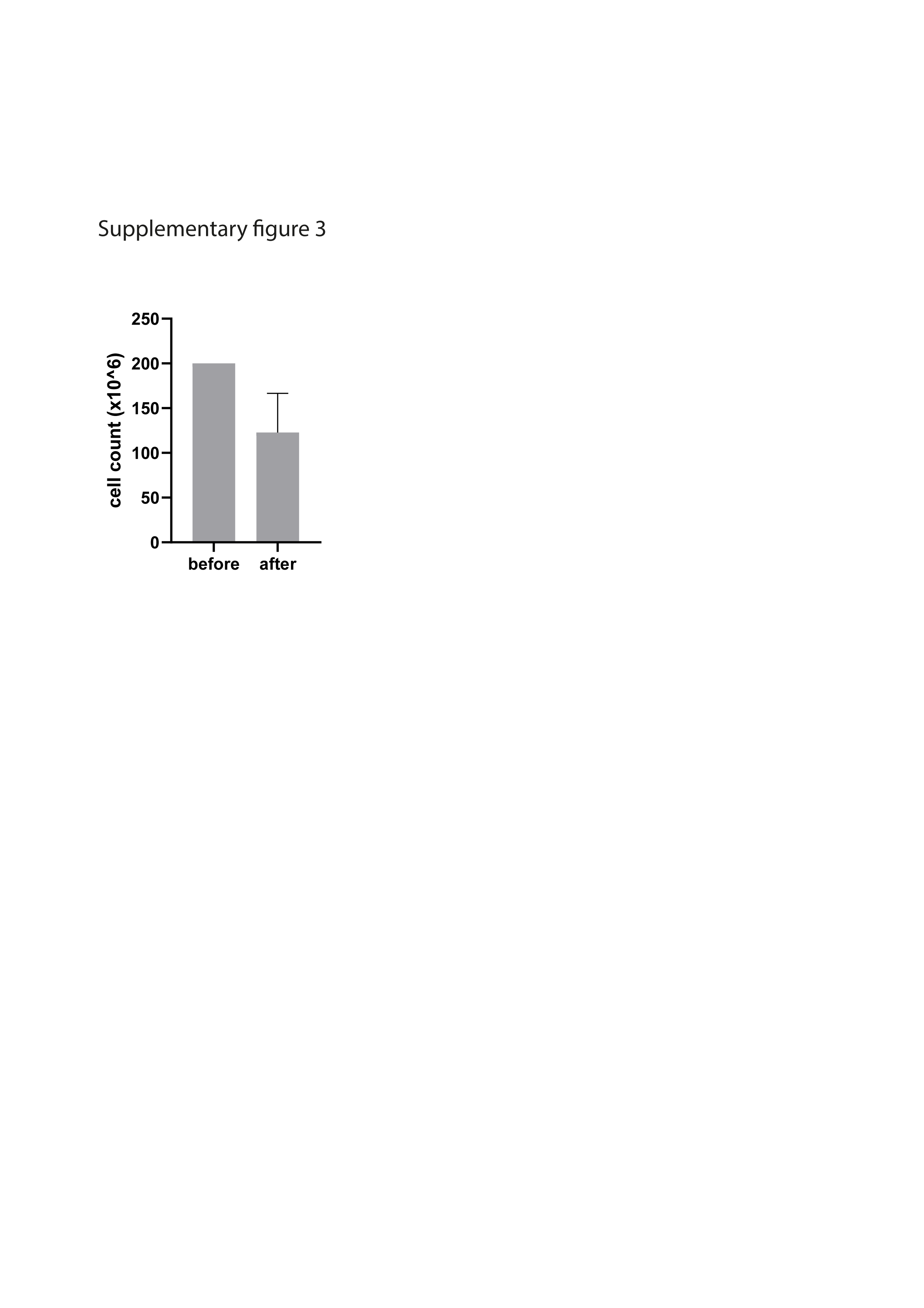

Supplement: Supplementary Figure 3 — Loss of cell number after EP. Cell count before and after EP. 200x10^6 cells were electroporated for each sample. [file Image_3.tif]

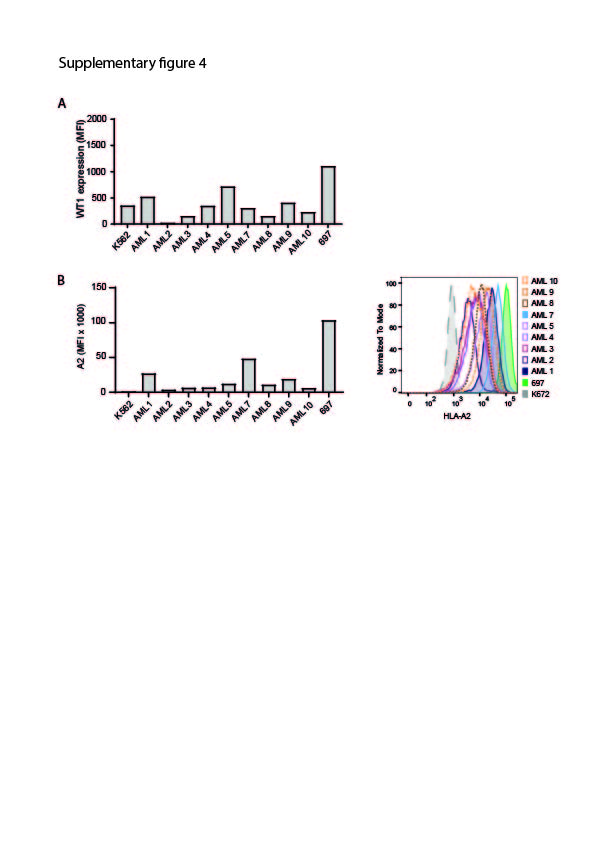

Supplement: Supplementary Figure 4 — WT1 and HLA-A2 expression by primary AML samples. All obtained AML samples were measured using flow cytometry. (A) Mean fluorescent intensity (MFI) of intracellular WT1 levels of the different primary pediatric AML samples. WT1 positive AML samples were selected for the killing assay (figure 5) (B) MFI of HLA-A2 levels expressed in bars (left) or histogram (right) to show variable expression although selected from the database on genetically positive levels of A2. [file Image_4.jpeg]
